# The Linkage Phase of the Polymorphism KCNH2-K897T Influences the Electrophysiological Phenotype in hiPSC Models of LQT2

**DOI:** 10.3389/fphys.2021.755642

**Published:** 2021-12-16

**Authors:** Lettine van den Brink, Karina O. Brandão, Loukia Yiangou, Albert Blanch-Asensio, Mervyn P. H. Mol, Christine L. Mummery, Arie O. Verkerk, Richard P. Davis

**Affiliations:** ^1^Department of Anatomy and Embryology, Leiden University Medical Center, Leiden, Netherlands; ^2^Department of Applied Stem Cell Technologies, University of Twente, Enschede, Netherlands; ^3^Department of Medical Biology, Amsterdam UMC, Amsterdam, Netherlands

**Keywords:** long QT syndrome type 2, disease modeling, human pluripotent stem cell-derived cardiomyocytes, isogenic, genetic modifier, cardiac electrophysiology, arrhythmia, hERG

## Abstract

While rare mutations in ion channel genes are primarily responsible for inherited cardiac arrhythmias, common genetic variants are also an important contributor to the clinical heterogeneity observed among mutation carriers. The common single nucleotide polymorphism (SNP) KCNH2-K897T is associated with QT interval duration, but its influence on the disease phenotype in patients with long QT syndrome type 2 (LQT2) remains unclear. Human induced pluripotent stem cells (hiPSCs), coupled with advances in gene editing technologies, are proving an invaluable tool for modeling cardiac genetic diseases and identifying variants responsible for variability in disease expressivity. In this study, we have used isogenic hiPSC-derived cardiomyocytes (hiPSC-CMs) to establish the functional consequences of having the KCNH2-K897T SNP in *cis*- or *trans*-orientation with LQT2-causing missense variants either within the pore-loop domain (KCNH2^A561T/WT^) or tail region (KCNH2^N996I/WT^) of the potassium ion channel, human ether-a-go-go-related gene (hERG). When KCNH2-K897T was on the same allele (*cis*) as the primary mutation, the hERG channel in hiPSC-CMs exhibited faster activation and deactivation kinetics compared to their *trans*-oriented counterparts. Consistent with this, hiPSC-CMs with KCNH2-K897T in *cis* orientation had longer action and field potential durations. Furthermore, there was an increased occurrence of arrhythmic events upon pharmacological blocking of hERG. Collectively, these results indicate that the common polymorphism KCNH2-K897T differs in its influence on LQT2-causing *KCNH2* mutations depending on whether it is present in *cis* or *trans*. This study corroborates hiPSC-CMs as a powerful platform to investigate the modifying effects of common genetic variants on inherited cardiac arrhythmias and aids in unraveling their contribution to the variable expressivity of these diseases.

## Introduction

Inherited primary arrhythmia syndromes are one of the most common causes of cardiovascular disease-related death among young people (<40 years) (Isbister and Semsarian, 2019). In long QT syndrome (LQTS), types 1–3 (LQT1–3) are the most predominant (Gnecchi et al., 2021). These are caused by primary mutations in the ion channel genes *KCNQ1*, *KCNH2*, and *SCN5A*, respectively, and result in a delayed ventricular repolarization, thereby predisposing patients to malignant arrhythmia and risk of sudden cardiac death. While the growing body of identified causative genes has increased our understanding of the disease (Adler et al., 2020), variable expressivity and incomplete penetrance still complicate the risk stratification and clinical management of patients with LQTS (Giudicessi and Ackerman, 2013). This heterogeneity is due to both environmental and genetic factors (Bezzina et al., 2015). It is widely recognized that besides the location and type of the primary mutation, the presence of additional common genetic variants can alter the disease expressivity (Napolitano et al., 2018; Schwartz et al., 2018).

While genome-wide association studies (GWASs) and individual genome sequencing have identified a number of potential genetic modifiers of LQTS (Lahrouchi et al., 2020; Walsh et al., 2020), establishing the contribution of these modifiers (or non-Mendelian variants) to the disease phenotype is challenging, since their effects are smaller than that of a disease-causing mutation (van den Brink et al., 2020b). Human induced pluripotent stem cells (hiPSCs), which are generated by reprogramming somatic cells from individuals (Takahashi et al., 2007), offer an approach to address this issue. These cells can be differentiated *in vitro* into cardiomyocytes (hiPSC-CMs) and are capable of replicating aspects of genetic disease phenotypes observed in the patients (Brandão et al., 2017; Garg et al., 2018). Combining this with advances in gene targeting technology, it has opened the door to validate variants as genetic modifiers of the disease phenotype and scrutinize their mechanism of action. Indeed, recent studies using these two techniques have identified novel modifying variants responsible for differences in the LQTS phenotype observed in patients with the same primary Mendelian variant (Chai et al., 2018; Lee et al., 2021).

The common single nucleotide polymorphism (SNP) rs1805123 (A → C) in *KCNH2* has a minor allele frequency (MAF) of ∼24% in populations of European descent (Bezzina et al., 2003) and leads to the substitution of lysine for threonine at residue 897 (K897T) in the potassium ion channel human ether-a-go-go-related gene (hERG), which is responsible for conducting the rapid component of the delayed rectifier potassium current (I_Kr_). While KCNH2-K897T has been identified as an intragenic modifier in some patients with LQT2, reports on its phenotypic impact and its effect on QT interval duration are discordant. Cohort- and family-based studies have classified KCNH2-K897T either as a protective or exacerbating modifier of the LQT2 phenotype (Crotti et al., 2005; Zhang et al., 2008; Nof et al., 2010; Jenewein et al., 2018). Similarly, *in vitro* studies using either heterologous expression systems or hiPSC-CMs have reported discrepant biophysical effects of K897T on hERG channel activity. In some cases, K897T has been reported to impact I_Kr_ by altering the gating properties of the channel (e.g., decelerated deactivation and inactivation, negative shift in voltage dependency), reducing I_Kr_ density, or by inhibition through Akt-mediated phosphorylation (Paavonen et al., 2003; Gentile et al., 2008; Nof et al., 2010). In contrast, other studies have observed accelerated activation and deactivation kinetics and no effects on current densities (Bezzina et al., 2003; Terrenoire et al., 2013). Furthermore, if these non-Mendelian variants occur in the same gene as the primary mutation, then phenotypic effects can differ depending on whether the variant is on the same (*cis*) or opposite (*trans*) allele (Giudicessi and Ackerman, 2013; Coll et al., 2018).

Therefore to investigate whether the chromosomal phase of KCNH2-K897T could also influence the functional consequences of primary *KCNH2* mutations, we took advantage of isogenic hiPSC models that were genetically edited to harbor heterozygous LQT2-causing missense variants either within the pore-loop domain (KCNH2^A561T/WT^) or cytoplasmic tail region (KCNH2^N996I/WT^) (Brandão et al., 2020). Functional comparisons revealed differences in the biophysical properties of hERG between the *cis*- and *trans*-oriented hiPSC-CM disease models. When KCNH2-K897T was present on the same allele as the primary mutation, the hiPSC-CMs exhibited faster activation and deactivation kinetics compared to their *trans*-linked counterparts. This was also reflected in longer action potential (AP) and field potential (FP) durations (FPDs). Finally, the LQT2 hiPSC-CMs with KCNH2-K897T in *cis* exhibited an increased arrhythmogenic response to the hERG channel blocker, E4031. This study demonstrates how genetically matched hiPSC-CM disease models can facilitate investigations into the functional impact of genetic modifiers including how the linkage phase can affect disease severity.

## Results

### Characterization of Isogenic KCNH2 Disease hiPSC Lines With K897T in *cis* and *trans* With the Risk Allele

**FIGURE 1:**
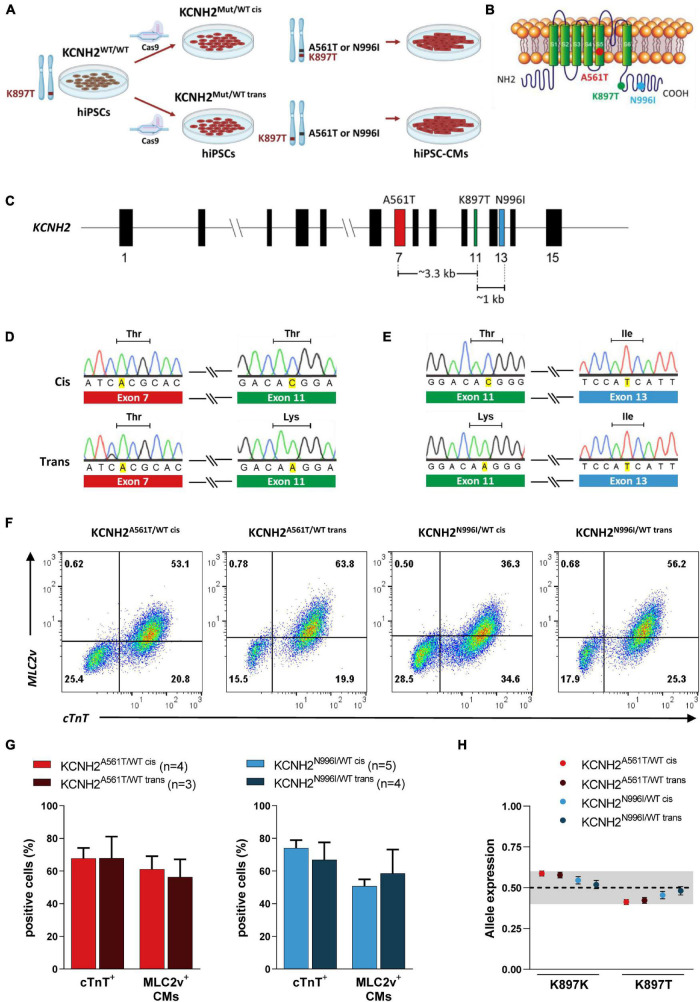
Genotyping of isogenic human induced pluripotent stem cell (hiPSC) lines with *KCNH2* mutations and their differentiation into cardiomyocytes. **(A)** Schematic outlining the generation of isogenic hiPSC-CMs harboring KCNH2-K897T either in *cis* or *trans* orientation with long QT syndrome type 2 (LQT2) missense mutations, p.A561T or p.N996I. **(B)** Structure of *KCNH2*-encoded ion channel, hERG, indicating the approximate location of the primary mutations and K897T. **(C)** Structure of the *KCNH2* genomic locus displaying the exons containing the missense mutations and the genomic distance between them. Exon numbers are indicated. **(D,E)** Sanger sequencing of gene-edited hiPSC lines KCNH2^A561T/WT^ **(D)** and KCNH2^N996I/WT^ **(E)** harboring KCNH2-K897T either in *cis* (top panel) or *trans* (bottom panel) orientation. Only the p.A561T and p.N996I mutated alleles and their corresponding K897T region of the sequences are shown. **(F)** Representative flow cytometry plots showing cardiac troponin T (cTnT) and myosin light chain 2v (MLC2v) expression in hiPSC-CMs at differentiation day 21 + 7. Numbers inside the plots are percentage of cells within the gated region. **(G)** Average percentage of hiPSC-CMs (cTnT^+^), and the proportion of ventricular-like (MLC2v^+^) cardiomyocytes within the hiPSC-CM population for the KCNH2^A561T/WT^ (left panel) and KCNH2^N996I/WT^ (right panel) cell lines. Values (n) indicate the number of differentiations analyzed. **(H)** Quantification of *KCNH2* expression specifically from the K897K and K897T alleles in the differentiated hiPSC lines and displayed as fractional abundance, with the shaded area indicating the region close to 0.5 (0.4–0.6). KCNH2^A561T/WT cis^ and KCNH2^A561T/WT trans^: *n* = 3 replicates; KCNH2^N996I/WT cis^ and KCNH2^N996I/WT trans^: *n* = 6 replicates. Error bars are the Poisson 95% CI.

To establish whether the biophysical effect of the KCNH2-K897T polymorphism on mutant hERG channel activity differed depending on whether the amino acid change was on the same or different subunit to a trafficking-deficient mutation, we made use of genetically-matched hiPSC lines bearing primary LQT2-causing *KCNH2* mutations. Previously, we introduced missense variants either within the pore-loop domain (A561T) or cytoplasmic tail region (N996I) in heterozygosity into a control hiPSC line (Brandão et al., 2020; Figures 1A,B). Subsequent genotyping of the hiPSC lines used in this study confirmed that the K897T polymorphism was present on the edited allele (KCNH2^A561T/WT cis^ and KCNH2^N996I/WT cis^) (Figures 1C-E and Supplementary Figure 1A). Further screening also identified edited hiPSC lines with the K897T polymorphism on the opposite allele to the primary *KCNH2* mutations (KCNH2^A561T/WT trans^ and KCNH2^N996I/WT trans^) (Figures 1D,E). These hiPSC lines were confirmed to have a normal G-band karyotype and to express stem cell markers such as SOX2 (SRY-box transcription factor 2), OCT-4 (POU class 5 homeobox 1), NANOG, and SSEA4 (stage-specific embryonic antigen-4) (Supplementary Figures 1B–E).

All four hiPSC lines efficiently differentiated into cardiomyocytes, with an average 67–74% of the cells (from three to five differentiations) expressing the pan-cardiomyocyte marker cardiac troponin T (cTnT) (Figures 1F,G). Similar percentages of hiPSC-CMs for each of the lines also expressed the ventricular cardiomyocyte marker, myosin light chain 2v (MLC2v; ∼50–60%) (Figures 1F,G). Additionally, hiPSC-CMs from all the four lines showed balanced allelic expression of the *KCNH2* transcript (Figure 1H).

### hERG Channel Kinetics in LQT2 hiPSC-CMs Is Influenced by the Linkage Phase of the K897T Polymorphism

**FIGURE 2:**
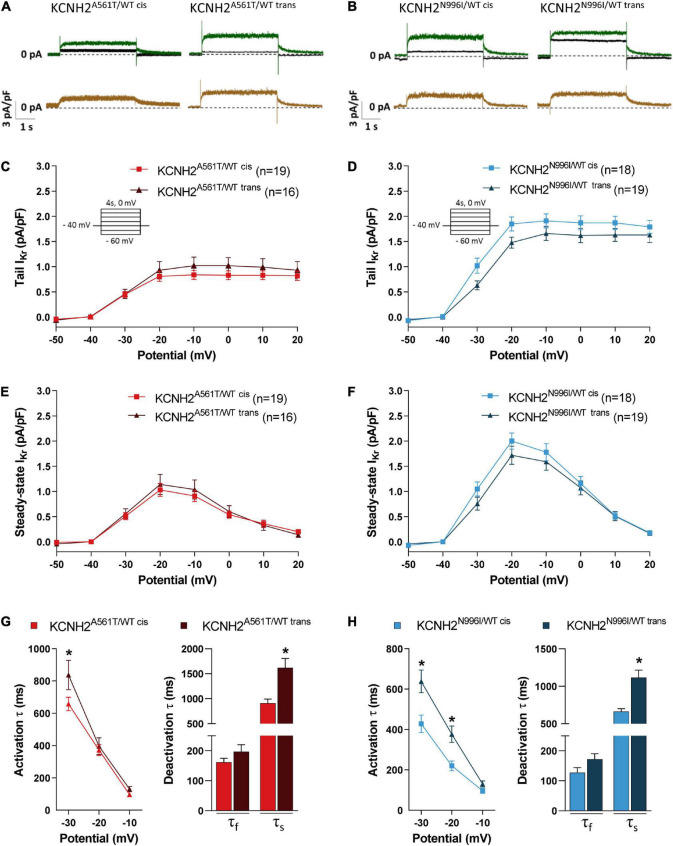
I_Kr_ properties in KCNH2^A561T/WT^ and KCNH2^N996I/WT^ hiPSC-CMs with KCNH2-K897T either in *cis* or *trans* orientation. **(A,B)** Representative current traces elicited upon 4 s depolarizing steps to –10 mV from a holding potential of –40 mV before (green lines) and after (black lines) application of 5 μM E4031 in isogenic sets of KCNH2^A561T/WT^ **(A)** and KCNH2^N996I/WT^ **(B)** hiPSC-CMs. The resulting E4031-sensitive traces are indicated in the bottom panel (brown lines). **(C–F)** Average current-voltage relationships for tail I_Kr_ current **(C,D)** and steady-state **(E,F)** densities in the indicated hiPSC-CMs. All the hiPSC-CMs activated upon depolarization reached a maximum steady-state current at –20 mV which decreased at more positive potentials due to the onset of inactivation. Inset: voltage protocol. Values (n) indicate the number of single hiPSC-CMs analyzed from three or four differentiations. **(G,H)** Average activation (left panel) and fast (τ_f_) and slow (τ_s_) deactivation (right panel, by stepping back from depolarizing step 0 mV to –40 mV holding potential) in KCNH2^A561T/WT^ **(G)** and KCNH2^N996I/WT^ **(H)** hiPSC-CMs. For KCNH2^A561T/WT cis^ and KCNH2^A561T/WT trans^: activation, *n* = 19 and *n* = 16; deactivation, *n* = 16 and *n* = 10 from three differentiations; for KCNH2^N996I/WT cis^ and KCNH2^N996I/WT trans^: activation *n* = 17 and *n* = 19; deactivation *n* = 17 and *n* = 19 from three or four differentiations for each cell line. *indicates statistical significance between *cis*-*trans* pairs [activation: –30 mV, KCNH2^A561T/WT^, *p* = 0.02; KCNH2^N996I/WT^, *p* < 0.001; –20 mV, KCNH2^N996I/WT^, *p* = 0.01; slow deactivation: KCNH2^A561T/WT^, *p* = 0.001; KCNH2^N996I/WT^, *p* < 0.001; the two-way ANOVA with the Sidak’s multiple comparison (activation) or the unpaired Student’s *t*-test (deactivation)].

To evaluate whether the linkage phase of the K897T polymorphism to the primary *KCNH2* variants A561T and N996I resulted in differences in I_Kr_, the current density and gating properties were determined in single hiPSC-CMs. Examples of current traces are shown in Figures 2A,B. As expected, I_Kr_ density in the disease lines was smaller compared to KCNH2^WT/WT^ hiPSC-CMs (Supplementary Figure 2A). For example, the maximal tail current density for KCNH2^WT/WT^ was 3.3 ± 0.2 pA/pF (*n* = 16), while it was 0.8 ± 0.1 pA/pF (*n* = 19), and 1.9 ± 0.1 pA/pF (*n* = 18) for KCNH2^A561T/WT cis^ or KCNH2^N996I/WT cis^, respectively. Tail and steady-state I_Kr_ densities did not differ significantly between the *cis* and *trans* pairs for KCNH2^A561T/WT^ or KCNH2^N996I/WT^ at any voltage step (Figures 2C–F). Furthermore, voltage dependency of activation was also similar between the pairs as indicated by the overlapping normalized tail current curves (Supplementary Figures 2B,C).

However, kinetics analysis revealed significant faster channel kinetics for the hiPSC-CMs harboring K897T on the mutant allele, namely KCNH2^A561T/WT cis^ and KCNH2^N996I/WT cis^ (Figures 2G,H). For example, I_Kr_ activation (τ) at –30 mV was 21 and 33% faster for the KCNH2^A561T/WT cis^ and KCNH2^N996I/WT cis^ compared to their respective *trans* counterparts. The slow component (τ_*slow*_) of the I_Kr_ deactivation obtained from the tail current decay also was significantly faster when K897T was in *cis* (Figures 2G,H). Finally, while the fast component (τ_*fast*_) of deactivation was on average faster when K897T was on the same allele as the mutation, this did not reach significance for either of the *KCNH2* variant pairs.

Taken together, this data indicated that while I_Kr_ current density and voltage dependency of activation in the *KCNH2* variant lines were not influenced by which allele carried the K897T polymorphism, when it was on the same allele as the disease variant, an acceleration in activation and deactivation kinetics were observed. The changes in (de)activation kinetics may have a direct effect on the AP and contribute to changes in the minimum diastolic potential (mDP) in the final phase of diastole of spontaneously active hiPSC-CMs, and thereby consequently affect channel gating and permeation of other voltage-gated ion channels (Verkerk and Wilders, 2021).

### Co-localization of KCNH2-K897T With the Primary Mutation Prolongs the Repolarization Period in hiPSC-CMs

**FIGURE 3:**
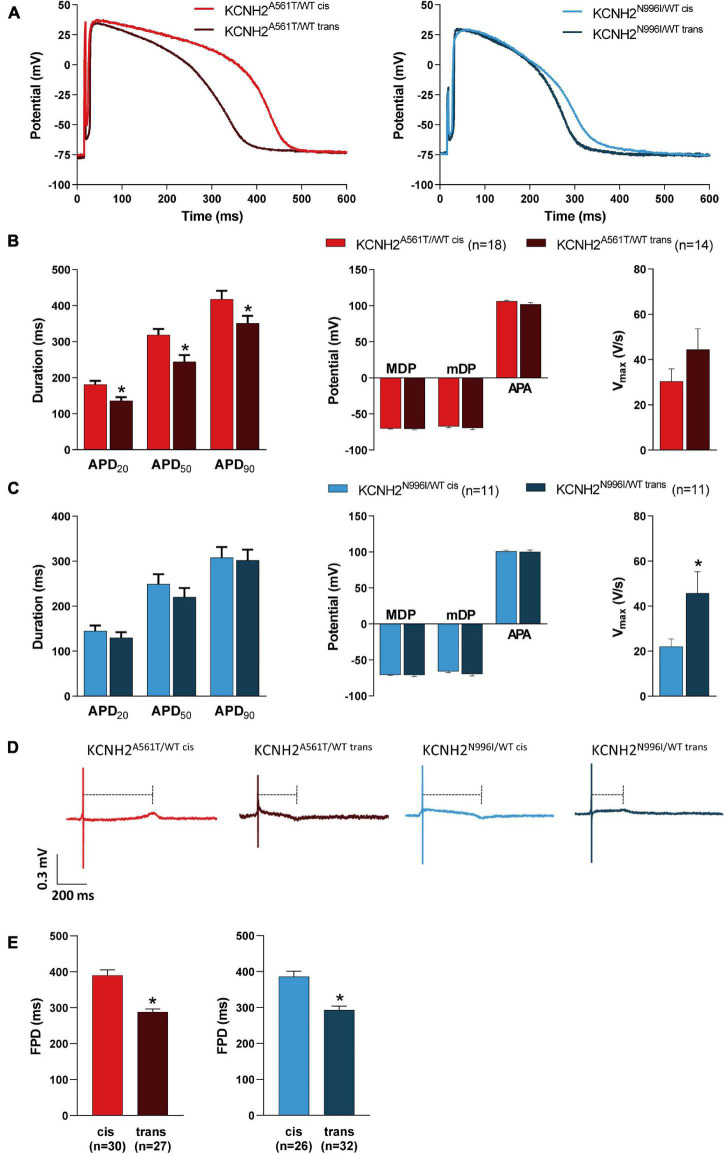
Electrophysiological characteristics of KCNH2^A561T/WT^ and KCNH2^N996I/WT^ hiPSC-CMs with KCNH2-K897T either in *cis* or *trans* orientation. **(A)** Representative AP traces of the pairs of KCNH2^A561T/WT^ (left panel) and KCNH2^N996I/WT^ (right panel) hiPSC-CMs measured at 1 Hz. **(B,C)** Average APD_20_, APD_50_, APD_90_, MDP, mDP, APA, and V_*max*_ at 1 Hz for hiPSC-CM clusters for both KCNH2^A561T/WT^ **(B)** and KCNH2^N996I/WT^ **(C)** isogenic pairs. Values (n) refer to the number of individual hiPSC-CM clusters from two or three differentiations for each cell line. * indicates statistical significance [for panel **(B)**, APD_20_ *p* = 0.003, APD_50_ *p* = 0.006, APD_90_ *p* = 0.04; for panel **(C)**, V_*max*_ *p* = 0.03; unpaired Student’s *t*-test]. **(D)** Representative MEA traces recorded at 0.8 Hz in the indicated hiPSC-CM monolayers. Dashed lines indicate the FPD. **(E)** Average FPD at 0.8 Hz for KCNH2^A561T/WT^ (left panel) and KCNH2^N996I/WT^ (right panel) isogenic pairs. Values (n) indicate the number of independent recordings analyzed from three or four differentiations. *indicates statistical significance [KCNH2^A561T/WT^ and KCNH2^N996I/WT^ *p* < 0.0001; unpaired Student’s *t*-test].

Subsequently, we investigated whether the allele carrying the K897T polymorphism also altered AP parameters in the mutant lines. AP recordings were obtained from both the spontaneously beating and paced clusters of CMs (10–20 cells; Supplementary Figure 3A), with representative APs paced at 1 Hz shown in Figure 3A. As expected, the four LQT2 hiPSC-CM lines all exhibited prolonged AP durations (APDs) and, consequently, the number of cells able to follow at 4 Hz pacing was reduced compared to KCNH2^WT/WT^ hiPSC-CMs (Supplementary Figures 3B,C). For the KCNH2-A561T lines at 1 Hz pacing, the APD at 20, 50, and 90% of repolarization (APD_20_, APD_50_, and APD_90_) was significantly prolonged when the K897T polymorphism was in *cis* compared to when it was not present on the risk allele (Figure 3B and Supplementary Table 1). Measurements from unpaced KCNH2^A561T/WT^ hiPSC-CMs displayed similar differences in APD (Supplementary Figures 4A,B). The difference in APD_90_ between the KCNH2^A561T/WT^ hiPSC-CMs was also present at slower pacing frequencies (Supplementary Figure 3B). In agreement with the shorter APD, a greater proportion of KCNH2^A561T/WT trans^ CMs could be paced at 2 Hz or faster (Supplementary Figure 3C). While paced KCNH2^N996I/WT^ hiPSC-CM clusters with the polymorphism in either allele showed no differences in APD or ability to follow pacing at ≤ 4 Hz (Figure 3C and Supplementary Figure 3), the APD was prolonged in spontaneously beating KCNH2^N996I/WT cis^ hiPSC-CMs (Supplementary Figure 4C and Supplementary Table 1).

In agreement with the faster I_Kr_ kinetics observed in the LQT2 hiPSC-CMs where K897T was in *cis* with the mutation (Figures 2G,H), these lines when measured spontaneously also had a more depolarized mDP and faster cycle length compared to their *trans* counterparts. However the latter was only significant for the pair of KCNH2^A561T/WT^ lines (Supplementary Figure 4 and Supplementary Table 1). It is likely that these differences contributed to the significantly lower AP upstroke velocity (V_*max*_) observed in the KCNH2^A561T/WT cis^ and KCNH2^N996I/WT cis^ hiPSC-CMs compared to their respective *trans* counterparts (Supplementary Figures 4B,C and Supplementary Table 1). Differences in mDP were not observed when the cells were paced because the external stimulus enabled AP initiation in a more hyperpolarized state (Figures 3A–C and Supplementary Table 1). In general, no significant differences in maximum diastolic potential (MDP) or AP amplitude (APA) were observed in either *cis*-*trans* hiPSC-CM pairs for the two *KCNH2* mutations (Figures 3B,C, Supplementary Figures 4B,C, and Supplementary Table 1), though APA was smaller in spontaneous beating KCNH2^A561T/WT trans^ compared to KCNH2^A561T/WT cis^ hiPSC-CMs (Supplementary Figure 4B and Supplementary Table 1).

We also investigated using multi-electrode arrays (MEAs) the impact of the allele orientation of the K897T polymorphism on the *KCNH2* mutations A561T and N996I in FP recordings of hiPSC-CM monolayers. To optically pace the hiPSC-CMs, the cells were transfected with Channelrhodopsin-2 (ChR2) messenger RNA (mRNA) (Supplementary Figure 5A). This did not affect the intrinsic beat rate or FPD of the hiPSC-CMs (Supplementary Figures 5B,C). Representative traces of hiPSC-CM monolayers paced at 0.8 Hz are shown in Figure 3D. All the four *KCNH2* mutant lines exhibited a prolongation at multiple pacing frequencies (0.5–2.0 Hz) compared to KCNH2^WT/WT^ hiPSC-CMs (Supplementary Figure 5D). Consistent with the AP measurements, FPD was prolonged when the K897T polymorphism was in *cis* (Figures 3D,E). The difference in FPD between the *cis* and *trans* pairs of hiPSC-CMs was also apparent at a range of pacing frequencies (0.5–1.5 Hz), with the longer FPD of the K897T *cis*-oriented hiPSC-CMs also meaning fewer monolayers than their *trans* counterparts could be paced at ≥1.5 Hz (Supplementary Figures 5D,E).

Collectively, these findings indicate that the differences in I_Kr_ kinetics are reflected in either the AP or FP characteristics of the hiPSC-CMs, with the lines in which KCNH2-K897T was in *cis* with the primary mutation having more prolonged APDs and FPDs compared to their *trans* counterparts.

### Long QT Syndrome Type 2 hiPSC-CMs With KCNH2-K897T in *cis* Exhibit an Increased Arrhythmogenic Response

**FIGURE 4:**
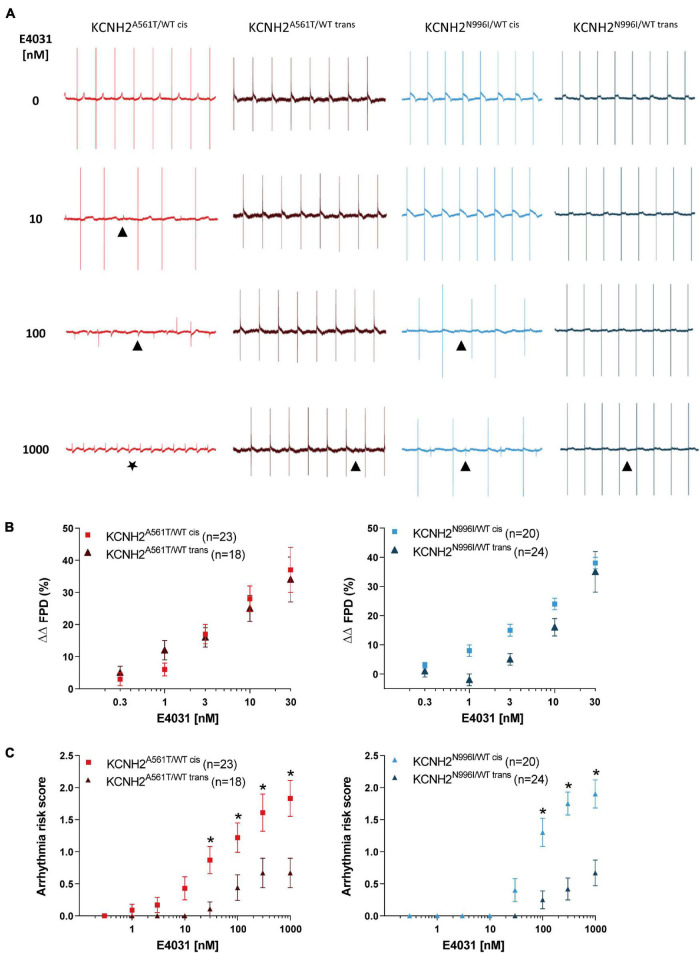
Effect of E4031 on FPD and arrhythmogenesis in KCNH2^A561T/WT^ and KCNH2^N996I/WT^ hiPSC-CMs. **(A)** Representative MEA traces recorded at 0.8 Hz in the indicated hiPSC-CM monolayers before and after cumulative addition of E4031 up to 1,000 nM. Symbols indicate examples of the different arrhythmia-like events observed: (▲) abnormal repolarization; (★) fibrillation. **(B)** Percent change in FPD relative to baseline and corrected for vehicle (ΔΔFPD) upon cumulative addition of E4031 in the KCNH2^A561T/WT^ (left panel) and KCNH2^N996I/WT^ (right panel) pairs of hiPSC-CMs. **(C)** Estimated arrhythmic risk score for the KCNH2^A561T/WT^ (left panel) and KCNH2^N996I/WT^ (right panel) pairs of hiPSC-CMs with cumulative addition of E4031. * indicates statistical significance between *cis*-*trans* pairs [KCNH2^A561T/WT^ *p* < 0.02 and KCNH2^N996I/WT^ *p* < 0.001; two-way ANOVA with Sidak’s multiple comparison test]. For panels **(B,C)**, values (n) indicate the number of E4031-treated wells analyzed from three or four differentiations.

Finally, we investigated whether the K897T polymorphism when oriented in *cis* with primary *KCNH2* mutations also altered the response of the hiPSC-CMs to the hERG channel blocker, E4031. Representative FP traces of hiPSC-CMs optically paced at 0.8 Hz and measured with increasing concentrations of E4031 (0–1,000 nM) are shown in Figure 4A. Because up to half of the recordings for some lines could not be paced at ≥ 30 nM E4031 due to the occurrence of arrhythmic events (Supplementary Figure 6), changes in FPD were analyzed only up to this concentration. Addition of 3–30 nM E4031 resulted in FPD prolongation when compared to baseline values and corrected for vehicle (ΔΔFPD) for all the four disease lines (Figure 4B). The linkage phase of the K897T polymorphism did not influence the change in ΔΔFPD observed in response to the cumulative addition of E4031 for the KCNH2^A561T/WT^ lines or for the KCNH2^N996I/WT^ hiPSC-CMs at 30 nM E4031. Although there appeared to be a difference in response between the KCNH2^N996I/WT cis^ and KCNH2^N996I/WT trans^ hiPSC-CMs at lower concentrations of E4031, this was not significant and was most likely due to an acute prolongation in FPD in some KCNH2^N996I/WT trans^ hiPSC-CMs following the initial addition of the vehicle.

The E4031-induced arrhythmic events in the hiPSC-CM monolayers were classified in weighted categories [no arrhythmia; abnormal repolarization (early after depolarization (EAD)-like); fibrillation; quiescence] and a previously developed scoring system (Brandão et al., 2020) used to calculate the arrhythmogenic risk for each line. Arrhythmic events were detected in the KCNH2^A561T/WT cis^ hiPSC-CMs from 1 nM E4031, while for the KCNH2^A561T/WT trans^ hiPSC-CMs, these only started to occur at higher concentrations (≥30 nM; Supplementary Figure 6B). At the highest concentration of E4031 analyzed (1,000 nM), 6 out of 18 (33%) monolayers of KCNH2^A561T/WT trans^ hiPSC-CMs exhibited abnormal repolarizations, while ∼70% of KCNH2^A561T/WT cis^ hiPSC-CM recordings displayed arrhythmia-like events including ∼40% of recordings showing fibrillation or quiescence. This resulted in the KCNH2^A561T/WT cis^ hiPSC-CMs having a significantly increased arrhythmia risk score compared to KCNH2^A561T/WT trans^ hiPSC-CMs from 30 nM E4031 (Figure 4C). Similar findings were observed also with the KCNH2^N996I/WT^ lines, with 1,000 nM E4031 causing a larger percentage of arrhythmia-like events in hiPSC-CMs in which KCNH2-K897T was on the same allele as N996I (85 vs. 33%, *cis* and *trans* respectively) (Supplementary Figure 6B). Also, KCNH2^N996I/WT cis^ hiPSC-CMs were more susceptible to E4031-induced arrhythmic events from 100 nM (Figure 4C). Overall, this data indicates that while the chromosomal phase of the K897T polymorphism with primary *KCNH2* mutations does not influence changes in E4031-induced FPD, there was an increased susceptibility to E4031-induced arrhythmic events when the SNP was in *cis*.

## Discussion

Variability in disease phenotype is a common feature of inherited arrhythmic disorders such as LQTS and can even be present between siblings carrying the same primary disease-causing mutation (Giudicessi and Ackerman, 2013). A contributing factor to this variable disease expressivity is common genetic variants such as SNPs, either in the same LQTS-susceptibility gene or in other genes that modulate cardiac ion channel function (Giudicessi and Ackerman, 2013; Schwartz et al., 2018). Furthermore, when both the modifying SNP(s) and primary mutation are located in the same gene, then the linkage phase can also influence the disease phenotype (Amin et al., 2012). Establishing the contribution of common genetic variants to the overall disease phenotype remains a significant challenge due to small effect sizes, presence of additional polymorphisms, and/or genetic background of the patient (Napolitano et al., 2018; van den Brink et al., 2020b). However, recent studies combining hiPSC models of LQTS with gene editing have demonstrated that this approach can facilitate in determining the contribution common variants in modifying genes have on the disease phenotype and establish their mechanism of action (Chai et al., 2018; Lee et al., 2021).

In addition to these family-specific approaches, factors capable of influencing disease severity may also be identified through GWAS. The common SNP rs1805123, which leads to KCNH2-K897T, has been associated with QTc interval variability in population-based studies, with the general consensus being that it leads to a shortening of the QT interval under resting conditions (Pietilä et al., 2002; Bezzina et al., 2003; Gouas et al., 2005; Pfeufer et al., 2005; Newton-Cheh et al., 2007; Marjamaa et al., 2009). However, in patients with LQT2, results are discordant with the variant linked to both the prolonging and shortening the QTc interval (Crotti et al., 2005; Zhang et al., 2008; Nof et al., 2010; Jenewein et al., 2018). In this study, we investigated whether KCNH2-K897T can modify the electrophysiological phenotypes and susceptibility to drug-induced arrhythmias observed in two hiPSC models of *KCNH2* mutations known to cause LQT2. Explicitly, we have examined whether the chromosomal phase of this common polymorphism influenced the disease phenotype by using genetically-matched pairs of hiPSC-CMs in which K897T was either in *cis* or *trans* with the *KCNH2* primary mutations, A561T or N996I. Use of isogenic pairs was critical to exclude the influence of any other genetic-modifying variants possibly present in the hiPSC line. The hiPSC-CMs with KCNH2-K897T on the mutant allele (*cis*) presented longer APDs or FPDs and augmented susceptibility to arrhythmic events in response to E4031 compared to their *trans* counterpart.

Consistent with previous findings (Brandão et al., 2020), I_Kr_ density was smaller in KCNH2^A561T/WT^ hiPSC-CMs compared to KCNH2^N996I/WT^ hiPSC-CMs (Figures 2C,D). However, the chromosomal phase of KCNH2-K897T did not alter the I_Kr_ density further in either of these disease models, suggesting that the polymorphism does not influence intracellular trafficking of the hERG channels. A previous study of hiPSC-CMs from LQT3 mutation carriers also did not detect differences in I_Kr_ tail current density, regardless of whether KCNH2-K897T was present in heterozygosity or homozygosity (Terrenoire et al., 2013).

However, we did observe that the chromosomal phase of KCNH2-K987T impacted the time dependence of activation and deactivation. The accelerated activation kinetics in the *cis*-configured variant lines could contribute to a gain-of-function mechanism as it may cause a larger potassium current outflow during the depolarization phase. However, countering this, the accelerated deactivation time constant could contribute to a loss-of-function, as it decreases the potassium outflow during the repolarization phase resulting in prolongation. Given that the role of I_Kr_ is most prominent in the repolarization phase of a cardiac AP, changes in the deactivation time constant will dominate any activation time constant consequences. This will then lead to a net loss-of-function of the channel if KCNH2-K897T is present on the same allele as the primary mutation and a prolongation of the AP.

Indeed, patch clamp or MEA recordings demonstrated prolonged repolarization durations for the lines with KCNH2-K897T in *cis* orientation compared to their *trans* counterparts. As we expected any electrophysiological differences to be small, AP measurements were performed in clusters of hiPSC-CMs to reduce the variability caused by the larger seal-leak current that occurs per cell with single cells (Krogh-Madsen et al., 2005; Sheng et al., 2012). We and others have previously shown that performing electrophysiological measurements in such multicellular format enables differences in disease phenotype to be detected in hiPSC-CM models of LQT2 that were not uncovered in single cardiomyocytes (Brandão et al., 2020; Shah et al., 2020).

The accelerated slow component of deactivation in the LQT2 hiPSC-CMs harboring KCNH2-K897T on the mutant allele will also decrease the role of I_Kr_ in maintaining the diastolic potential in spontaneously active hiPSC-CMs, especially late during diastole (Doss et al., 2012). This was reflected in the spontaneously beating *cis*-oriented hiPSC-CMs exhibiting a less negative mDP immediately prior to the initiation of the AP. As a consequence of the more positive mDP, the maximal upstroke velocity was lower in these hiPSC-CMs, likely due to enhanced inactivation of Na^+^ channels (Verkerk and Wilders, 2021). The cells with KCNH2-K897T in *cis* configuration also exhibited a prolonged APD that was most prominent during spontaneous activity. As this prolongation was already apparent at APD_20_ and so outside the voltage range of I_Kr_ deactivation, the more pronounced mDP depolarization and the consequent effects on other voltage-gated ion channels may also contribute to the observed APD prolongation.

We also evaluated whether the chromosomal phase of K897T relative to the primary *KCNH2* mutations altered the frequency that drug-induced arrhythmogenic events occurred in the hiPSC-CM disease models. The blocking of hERG by E4031 revealed comparable FPD prolonging effects regardless of whether the polymorphism was in *cis* or *trans* configuration. However, a significant proportion of the LQT2 hiPSC-CMs in which KCNH2-K897T was also present on the risk allele could not be paced when E4031 was > 100 nM, while most of the *trans*-configured cells followed pacing at all the concentrations of E4031. Classifying and scoring the types of arrhythmic events detected indicated that the polymorphism in *cis* with the primary mutation resulted in a higher arrhythmogenic risk when E4031 was ≥ 30 nM. These differences in arrhythmogenic susceptibility might arise from the altered I_Kr_ channel kinetics as faster deactivation properties have been proposed to aggravate the LQTS phenotype following exposure to hERG channel blockers (Romero et al., 2015).

There have been conflicting reports of the impact of the KCNH2-K897T polymorphism on the disease phenotype in patients with LQT2 with identified primary *KCNH2* mutations. Several studies found LQT2 family members to only be symptomatic when they co-inherited the polymorphism (Crotti et al., 2005; Nof et al., 2010), while others observed less severe symptoms (Zhang et al., 2008; Jenewein et al., 2018). These discrepancies could be related to the type and location of the primary mutation, the presence of additional QT interval-modifying variants, or whether the disease-causing mutant is in *cis* or *trans* linkage with K897T. By using gene editing to introduce primary mutations into control hiPSC lines that are genetically defined, the variable effects that these genetic background differences introduce can be avoided. Furthermore, these models also enable the investigation of the potential differential impact that the linkage phase of the polymorphism might have with the primary *KCNH2* mutation.

Although only one clone for each genotype was included in this study, we previously examined two independently derived clones for each of the *KCNH2* mutant lines harboring KCNH2-K897T in *cis* and observed similar phenotypes in both the clonal lines (Brandão et al., 2020). In this study, we have investigated the effect of the linkage phase of K897T polymorphism for both the primary *KCNH2* mutations, with similar differences observed for both when KCNH2-K897T was on the same allele as the mutation and, thus, indicating that these are unlikely to be due to variants that have arisen spontaneously in culture or from nuclease-induced off-target effects. Sequencing of *KCNH2* in the control hiPSC line confirmed that no other amino acid-altering variants were present. Furthermore, *KCNH2* was equally expressed from both the alleles in the hiPSC-CMs from all the lines examined, indicating that non-coding variants were not contributing to the observed differences.

Follow-up studies in which the K897T variant is corrected in the cell lines would be valuable to determine whether this common polymorphism plays a protecting or exacerbating role in QTc prolongation and the consequential effect on disease severity in patients with LQT2. Likewise, additional molecular-based investigation into whether the polymorphism influences intracellular trafficking of either wild type or mutant hERG channels, for example, by epitope tags into *KCNH2* (Kanner et al., 2018), could provide further insight into the pathogenesis of LQT2. Finally, while all the hiPSC-CMs compared were culture age-matched and appeared to be of a comparable level of maturity based on MLC2v expression and similar MDP, the hiPSC-CMs are relatively immature with respect to ion channel properties compared to adult cardiomyocytes (Veerman et al., 2015).

In conclusion, we have demonstrated in hiPSC-CMs that the chromosomal phase of the KCNH2-K897T polymorphism with LQT2-associated mutations influences the biophysical properties of I_Kr_, prolonging the repolarization phase, and increasing drug-induced arrhythmogenicity when *cis*-configured. This study aids in advancing hiPSC-CMs as a model to gain insight into the genetic causes of variable disease expressivity in patients with congenital LQTS and help to refine risk stratification and precision medicine approaches for these diseases.

## Materials and Methods

### Linkage Determination of the K897T Polymorphism in the LQT2 hiPSC Lines

The KCNH2^A561T/WT^ and KCNH2^N996I/WT^ hiPSC lines were generated as previously described (Brandão et al., 2020). Sequencing of parental hiPSC KCNH2^WT/WT^ line [LUMC0020iCTRL; (Zhang et al., 2014)] determined the line was heterozygous for a substitution of lysine to threonine at position 897 of KCNH2 (K897T). Chromosomal phasing of the KCNH2-K897T polymorphism with the introduced LQT2 mutation was carried out on the resulting hiPSC clones (Supplementary Figure 1A). For KCNH2^A561T/WT^, this was performed using the One-Step PrimeScript RT-PCR (Takara Bio Incorporation, Kusatsu, Japan) on RNA isolated from hiPSC-CMs as described by the manufacturer; while for KCNH2^N996I/WT^, genomic DNA was isolated from the hiPSCs for PCR amplification (Supplementary Figure 1A). The resulting amplified products were cloned into pMiniT 2.0 [New England Biolabs (NEB), Ipswich, MA, United States] and genotyped by Sanger sequencing for both the primary mutation and the K897T polymorphism. The primers used are given in Supplementary Table 2. The previously characterized KCNH2^*A56*1T/WT^ and KCNH2^N996I/WT^ hiPSC lines [LUMC0020iHERG-03 and LUMC0020iHERG-01, respectively (Brandão et al., 2020)] were confirmed to have the KCNH2-K897T SNP in *cis* configuration with the primary mutations. Additional targeted clones, LUMC0020iHERG-06 (KCNH2^A561T/WT trans^) and LUMC0020iHERG-09 (KCNH2^N996I/WT trans^), were identified as harboring KCNH2-K897T in *trans* configuration with the primary mutations.

### Culture and Differentiation of hiPSCs

The hiPSCs were maintained in Essential 8 medium (Thermo Fisher Scientific, Waltham, MA, United States) on vitronectin (Thermo Fisher Scientific)-coated plates as previously described (Brandão et al., 2020). The hiPSCs were differentiated into cardiomyocytes using the Pluricyte Cardiomyocyte Differentiation kit (Ncardia, Leiden, Netherlands) according to the protocol of the manufacturer. After 20–21 days of differentiation, the hiPSC-CMs were dissociated and cryopreserved as previously described (van den Brink et al., 2020a; Campostrini et al., 2021). For replating, frozen hiPSC-CMs were thawed as described (Brandão et al., 2020) and replated in Medium C (Ncardia) with RevitaCell Supplement (1:100 dilution, Thermo Fisher Scientific). Media refreshments were performed 24 h thereafter and subsequently every 2–3 days. The hiPSC-CMs were evaluated 7–12 days after thawing, with the hiPSC-CMs age-matched in each assay (summarized in Supplementary Table 3).

### Karyotyping

The hiPSC lines were karyotyped by G-banding. Chromosome spreads of 20 metaphases per cell line were performed and analyzed by the Laboratory for Diagnostic Genome Analysis (Leiden University Medical Center).

### Flow Cytometric Analysis

Flow cytometry analysis of hiPSCs and hiPSC-CMs was performed as previously described (Brandão et al., 2020). The hiPSCs were incubated with the conjugated antibodies OCT-4-BV421 (1:25, Cat# 565644, BD Biosciences, Franklin Lakes, NJ, United States), SOX2-A488 (1:200, Cat# 53-9811-80, eBioscience, Waltham, MA, United States), or SSEA4-PE-vio770 (1:25, Cat# 130-105-081, Miltenyi Biotec, Bergisch Gladbach, Germany). 7–8 days post-thawing, hiPSC-CMs were fixed, permeabilized, and incubated with cTnT-Vioblue (1:11, Cat# 130-109-814, Miltenyi Biotec), and MLC2v-PE (1:11, Cat# 130-106-183, Miltenyi Biotec). A MacsQuant VYB flow cytometer (Miltenyi Biotec) was used for data acquisition and FlowJo software (FlowJo, Ashland, OR, United States) for data analysis.

### Immunofluorescence Analysis

The hiPSCs were plated in 24-well cell culture plates at 0.5 × 10^5^/cm^2^ and fixed 2 days later using 4% paraformaldehyde for 15 min. Fixed cells were permeabilized with phosphate buffer saline (PBS)/0.1% Triton X-100 (Sigma-Aldrich, Saint Louis, MO, United States) and blocked with 1% bovine serum albumin (Sigma-Aldrich)/0.05% Tween (Merck, Kenilworth, NJ, United States) in PBS. Cells were first incubated with NANOG (1:200, Cat# AF1997, R&D Systems, Minneapolis, MN, United States) and SSEA4 (1:200, Cat# SC59368, Santa Cruz Biotechnology, Santa Cruz, CA, United States) or with conjugated SOX2-A488 (1:200, Cat#53-9811-80, eBioscience). The primary antibodies to NANOG and SSEA4 were detected with AlexaFluor 555- (1:500, Cat#A21432, Thermo Fisher Scientific) and Alexa Fluor 488- (1:500, Cat#A-21202, Thermo Fisher Scientific) conjugated antibodies, respectively. The antibodies were diluted in permeabilization medium (medium B; Thermo Fisher Scientific) and incubated overnight at 4°C or 1.5 h at room temperature (RT) for the primary and secondary antibodies, respectively. Nuclei were labeled with 4′6-Diamidino-2-Phenylindole (DAPI) (0.3 μM). Images were captured using an EVOS FL Auto 2 Cell Imaging System (Thermo Fisher Scientific).

### ddPCR

RNA was extracted from hiPSC-CMs 7–8 days post-thawing using the NucleoSpin^®^ RNA Kit (Macherey-Nagel, Hoerdt, France) and treated with the DNA-free™ Kit (Thermo Fisher Scientific). 1 μg of RNA was transcribed into complementary DNA (cDNA) using the iScript™ cDNA Synthesis Kit (Bio-Rad, Hercules, CA, United States). Droplet digital PCR (ddPCR) was performed using a thermocycler, the Q200 AutoDG and QX200*™* Droplet Digital PCR System (Bio-Rad). Assays comprising of premixtures of a forward and reverse primer (18 μM each) with a fluorescein (FAM) or hexachlorofluorescein (HEX) conjugated hydrolysis probe (5 μM) were designed based on predefined criteria (Bell et al., 2018). Sequences for the primers and probes are given in Supplementary Table 2. The FAM-conjugated probe was designed to specifically bind the *KCNH2* wild-type sequence (K897K), while the HEX-conjugated probe recognized the *KCNH2* sequence harboring K897T. To improve target specificity, three or four locked nucleic acids were included per probe. Reactions (22 μl) were prepared with 2x Supermix for probes with no deoxyuridine triphosphate (Bio-Rad), 900 nM of each primer, 250 nM of each probe, and 3 μl cDNA digested with 4 U *Mse*I (NEB). Droplet generation, PCR amplification, and analysis were all performed according to the instructions of the manufacturer.

Allelic balance was determined with the QuantaSoft software (Bio-Rad) using the “Poisson Fractional Abundance Max” and “Poisson Fractional Abundance Min” values, which define the 95% CI around the estimate of allelic balance recorded under “Fractional Abundance.” Samples were classified as having a balanced allelic expression, if the values for each allele were within 0.5 ± 0.1 (Kamitaki et al., 2018).

### Patch-Clamp Electrophysiology

10 mm glass coverslips were coated with 1:100 Matrigel in DMEM-F12 (Thermo Fisher Scientific) for 2 h at 37°C before the thawed cells were seeded at 1.0–1.5 × 10^4^/cm^2^. Patch-clamp measurements were performed 7–12 days later using an Axopatch 200B Amplifier (Molecular Devices, San Jose, CA, United States) and digitized with a Digidata 1440A (Molecular Devices). Data acquisition, voltage control, and analysis were performed using pClamp10.7 software (Axon Instruments, Melbourne, Australia). Potentials were corrected for the calculated liquid junction potential (Barry and Lynch, 1991). AP and I_Kr_ sampling rates were 40 and 20 kHz, respectively. I_Kr_ traces were filtered with a low-pass Gaussian filter at 0.9 Hz. Glass capillary patch pipettes (World Precision Instruments, Sarasota, FL, United States) pulled from a Sutter P97 micropipette puller (resistance 1.8–2.5 MΩ) were used and series resistance was compensated for 80%. Cell membrane capacitance (pF) was estimated by dividing the decay time constant of the capacitive transient in response to 5 mV hyperpolarizing steps from −40 mV by series resistance. All the measurements were performed at 36.0 ± 0.5°C.

#### Voltage Clamp Measurements

I_Kr_ was measured in spontaneously contracting cells (7–10 days post-thawing) using ruptured patch clamp methodology in Tyrode’s solution containing (in mM): 140 NaCl, 5.4 KCl, 1.8 CaCl_2,_ 1.0 MgCl_2_, 5.5 glucose, and 5.0 HEPES; pH 7.4 (NaOH). Pipettes (resistance ∼2 MΩ) were filled with (in mM): 125 K-gluconate, 20 KCl, 5 K_2_-ATP, 10 HEPES, and 10 EGTA; pH 7.2 (KOH). I_Kr_ was activated by 4 s hyper- and depolarizing pulses from a holding potential of –40 mV, at a cycle length of 10 s, and in the presence of 5 μM nifedipine (Sigma-Aldrich). I_Kr_ was measured as a 5 μM E4031-sensitive current (Tocris, Bioscience, Bristol, England) by subtracting current traces before and after application of E4031. Current densities were analyzed by calculating delta pA at the end of the depolarizing voltage step (steady state) or upon stepping back to the holding potential (tail) and dividing by pF. Speed of I_Kr_ activation and deactivation was analyzed using monoexponential and biexponential fittings, respectively (Clampfit 10.7).

#### Current Clamp Measurements

APs were recorded using amphotericin-perforated patch-clamp methodology in spontaneously contracting two-dimensional clusters of hiPSC-CMs (10–20 cells), 7–12 days after replating, in Tyrode’s solution and using a pipette solution containing (in mM): 125 K-gluconate, 20 KCl, 5.0 NaCl, 0.44 amphotericin-B, and 10 HEPES (pH 7.2; KOH). APs were evoked at 0.2–4.0 Hz by 3 ms and 1.2–1.3x threshold current pulses through the patch pipette. The AP parameters (V_*max*_, mDP, MDP, APA, APD_20_, APD_50_, and APD_90_) were analyzed from at least 10 consecutive APs and averaged.

### Multi-Electrode Array Electrophysiology

For MEA experiments, 96-well Lumos MEA plates (M768-tMEA-96OPT, Axion Biosystems, Atlanta, GA, United States) were coated with human fibronectin (40 μg/ml, Alfa Aesar, Haverhill, MA, United States) for 1 h at 37°C and thawed hiPSC-CMs seeded onto the eight recording electrodes at a density of 4.4 × 10^5^/cm^2^. All the experiments were performed at 37°C in a 5% carbon dioxide (CO_2_) controlled environment, with the hiPSC-CMs (11–12 days post-thawing) equilibrated inside the device (Maestro Pro Multiwell MEA platform; Axion Biosystems) for 10 min prior to recording. FP traces were recorded at a sampling frequency of 12.5 kHz and digital low- and high-pass filters of 2,000 Hz Kaiser window and 0.1 Hz infinite impulse response (IIR), respectively.

#### *In vitro* Transcription

The coding sequence for ChR2 fused to enhanced yellow fluorescent protein (eYFP) was PCR amplified from the plasmid pLenti-EF1a-hChR2 (H134R)-EYFP-WPRE (#20942, Addgene, Watertown, MA, United States) using primers in which the 5′ primer incorporated the T7 polymerase promoter sequence (Supplementary Table 2). Following purification, the PCR product was digested with *Nde*I (NEB) to generate a 5′ overhang at the 3′ end of template. The digested product was gel extracted (Wizard SV Gel and PCR Clean-Up System; Promega, Madison, WI, United States) according to the instructions of the manufacturer and subsequently concentrated (>160 ng/μl) by ethanol precipitation. *In vitro* transcription (IVT) was performed using the INCOGNITO T7 ARCA 5mC- and Ψ-RNA Transcription Kit (Cellscript, Madison, WI, United States) following the instructions of the manufacturer, with the resulting mRNA precipitated overnight using LiCl. To the purified mRNA, a 5′ cap nucleotide and a 3′ poly(A) tail were sequentially added using the ScriptCap Cap 1 Capping System and A-Plus Poly(A) Polymerase Tailing Kit, respectively (Cellscript). The resulting capped and tailed mRNA was precipitated again using LiCl and quantified. The mRNA integrity was confirmed by electrophoresis using the Reliant RNA Precast Gel (Lonza, Basel, Switzerland).

#### Optical Pacing of hiPSC-CMs

At day 7 post-seeding, the hiPSC-CMs were transfected with *in vitro* transcribed ChR2-eYFP mRNA. The medium was refreshed 1 h prior to mRNA transfection (40–60 ng/well). The Lipofectamine™ Stem Transfection Reagent (Thermo Fisher Scientific) was first diluted (1:14) in Opti-MEM™ Reduced Serum Medium (Cat# 51985-026, Thermo Fisher Scientific). The ChR2 mRNA was then added to the lipofectamine mixture and incubated at RT for ∼10 min to form a liposome-mRNA complex before adding to the cells (15 μl per well). Untransfected controls were transfected with lipofection mix that did not contain the ChR2-eYFP mRNA. Media was refreshed ∼18 h post-transfection and then 3–4 days later before recording. 1 μM all-*trans* retinal (50 μl/well) (Sigma-Aldrich) was added to all the wells at least 1 h before baseline recordings at spontaneous beat rates. Spontaneous recordings were obtained from transfected and untransfected controls. At baseline and following E4031 addition, the transfected hiPSC-CMs were optically paced at frequencies ranging from 0.5 to 3.0 Hz using the Lumos™ Optical Stimulation System (Axion Biosystems). Settings for optical stimulation with 475 nm wavelength light are shown in Supplementary Table 4.

#### E4031 Treatment

E4031-induced effects on the hiPSC-CMs were evaluated by sequentially increasing the concentration (300 pM to 1 μM). 10 μm E4031 (Tocris Bioscience) dissolved in dimethyl sulfoxide (DMSO; Sigma-Aldrich) was serially diluted in Medium C (Ncardia). Each concentration was accomplished by the stepwise removal of 7 μl medium and addition of 7 μl diluted E4031 (3.5% replacement of the total volume in each well). The hiPSC-CMs were paced at 0.8 Hz for the entire duration, with the response to E4031 recorded for 3 min after an incubation period of 10 min. The maximum amount of DMSO present in the culture medium was 0.01%, with addition of the vehicle not significantly altering the FPD or inducing arrhythmic events in any of the lines.

Field potential duration and beat period were quantified over the whole recording time using the Cardiac Analysis Tool (Axion Biosystems). Signal-to-noise ratio was high and did not require filtering prior to analysis. No frequency correction was applied on the unpaced recordings, since this overestimated the rate-dependency of the FPD. Drug responses were normalized to their baseline measurement and corrected for their response to DMSO (ΔΔFPD). Arrhythmia-like events were classified and scored in weighted categories [no arrhythmia (score 0), abnormal repolarization/EAD like (score 2), fibrillation (score 3), and quiescence (score 4)] based on a previously developed scoring system (Brandão et al., 2020). Fibrillation was defined as a severe reduction in the FP amplitude, resulting in an almost complete abolishment of the Na^+^ current driven depolarization and accompanied with the presence of uncoordinated electrical activity. Quiescence was always preceded by abnormal repolarization and fibrillation.

### Statistical Data Analysis

All the data are presented as mean ± SEM. Statistical tests performed are indicated in the “Results” section or in the Figure legends. Comparisons of two KCNH2^Mut/WT^ lines to the KCNH2^WT/WT^ line (three groups) were performed using the one-way ANOVA followed by the Tukey’s multiple comparisons test for *post hoc* analysis. If one of the null hypotheses could be rejected, pairwise comparisons were conducted using the Student’s *t*-test (Goeman and Solari, 2011). Differences were considered statistically significant at *p* < 0.05. Analyses were conducted with GraphPad Prism 8 software (GraphPad, San Diego, CA, United States).

## Data Availability Statement

The original contributions presented in the study are included in the article/Supplementary Material, further inquiries can be directed to the corresponding author/s.

## Author Contributions

LB designed and conducted experiments, performed analysis, and wrote the manuscript. KB, LY, AB-A, and MM designed and conducted experiments. CM and AV provided scientific support and edited the manuscript. RD funded, conceived and supervised the project, wrote and edited the manuscript. All authors approved the submission of manuscript.

## Conflict of Interest

CM is a cofounder of Pluriomics B. V. (now Ncardia B. V.). The remaining authors declare that the research was conducted in the absence of any commercial or financial relationships that could be construed as a potential conflict of interest.

## Publisher’s Note

All claims expressed in this article are solely those of the authors and do not necessarily represent those of their affiliated organizations, or those of the publisher, the editors and the reviewers. Any product that may be evaluated in this article, or claim that may be made by its manufacturer, is not guaranteed or endorsed by the publisher.
