# Supplementary material for: The Linkage Phase of the Polymorphism KCNH2-K897T Influences the Electrophysiological Phenotype in hiPSC Models of LQT2
Source: Front Physiol. 2021 Dec 16;12:755642. doi: 10.3389/fphys.2021.755642 (PMC8726482; doi:10.3389/fphys.2021.755642)
Supplement: Supplementary file 1 [file Presentation_1.pdf]

Supplementary Material

SUPPLEMENTARY FIGURES

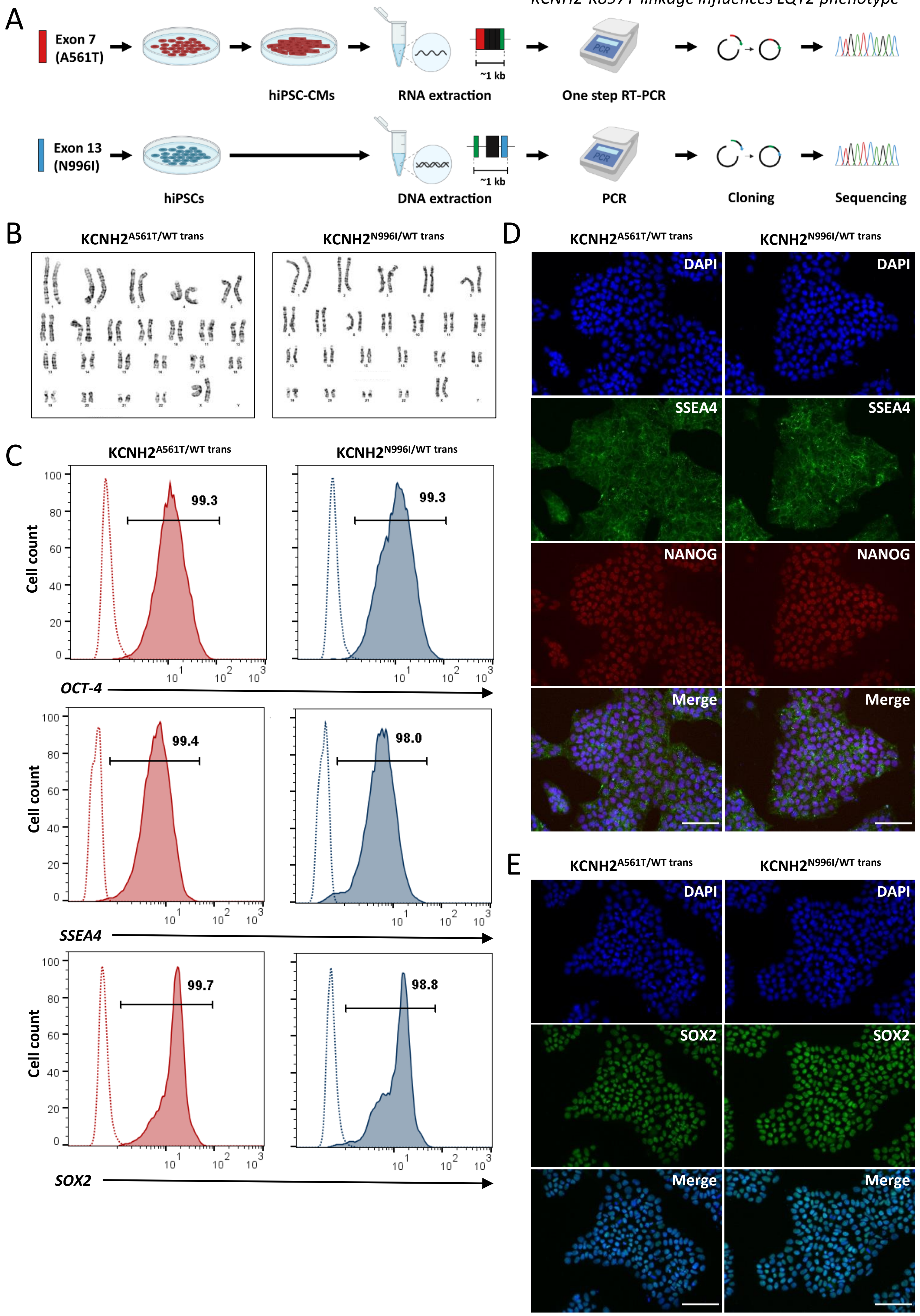
A
Exon 7
(A561T)
hiPSC-CMs
RNA extraction
~1 kb
One step RT-PCR
Exon 13
(N996I)
hiPSCs
DNA extraction
~1 kb
PCR
Cloning
Sequencing
B
KCNH2A561T/WT trans
KCNH2N996I/WT trans
C
KCNH2A561T/WT trans
KCNH2N996I/WT trans
Cell count
99.3
99.3
OCT-4
99.4
98.0
SSEA4
99.7
98.8
SOX2
D
KCNH2A561T/WT trans
KCNH2N996I/WT trans
DAPI
SSEA4
NANOG
Merge
E
KCNH2A561T/WT trans
KCNH2N996I/WT trans
DAPI
SOX2
Merge

FIGURE S1 | Characterization of the hiPSC lines KCNH2$^{A561T/WT}$ and KCNH2$^{N996I/WT}$ with KCNH2-K897T in *trans* to the primary mutation. (A) Schematic outlining the strategy to identify the KCNH2-K897T linkage phase from either cDNA or genomic DNA for the KCNH2$^{A561T/WT}$ and KCNH2$^{N996I/WT}$ lines respectively. **(B)** G-banding karyograms for the indicated hiPSC lines exhibited a normal 46, XX karyotype. **(C-E)** Flow cytometry **(C)** and immunofluorescence **(D-E)** analysis of the pluripotency markers OCT-4, SSEA4, SOX2, and NANOG in the indicated hiPSC lines. Dashed lines in histogram plots represent the control hiPSC population. Scale bar in images: 100 μm.

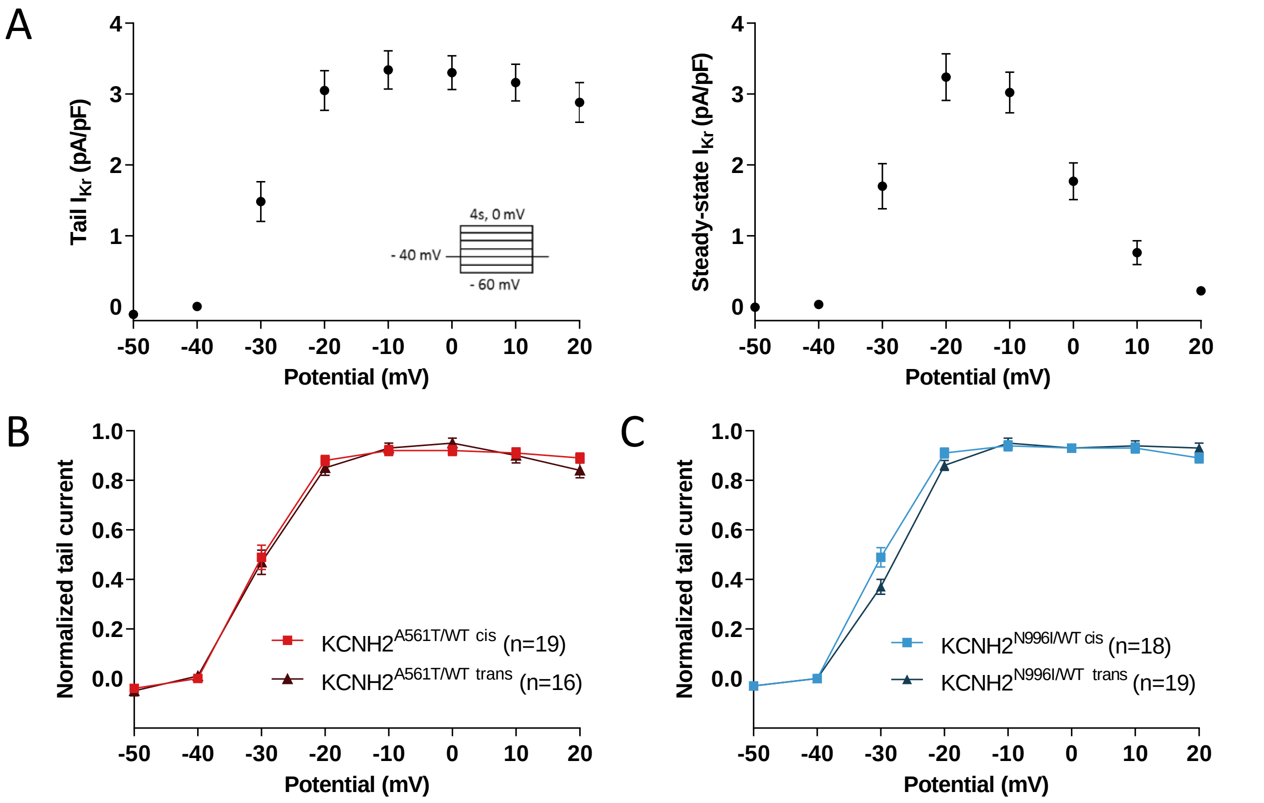

FIGURE S2 | I_{Kr} current characteristics in KCNH2$^{WT/WT}$ and KCNH2$^{Mut/WT}$ hiPSC-CMs. (A) Average current-voltage relationships for tail I_{Kr} current (left panel) and steady-state (right panel) densities in the KCNH2$^{WT/WT}$ hiPSC-CMs. Inset: voltage protocol. n=16 from four differentiations. **(B & C)** Average tail I_{Kr} current normalized to the maximal current following repolarization to holding potential -40 mV in KCNH2$^{A561T/WT}$ **(B)** and KCNH2$^{N996I/WT}$ **(C)** pairs of hiPSC-CMs. n, number of individual hiPSC-CMs analyzed from three or four differentiations.

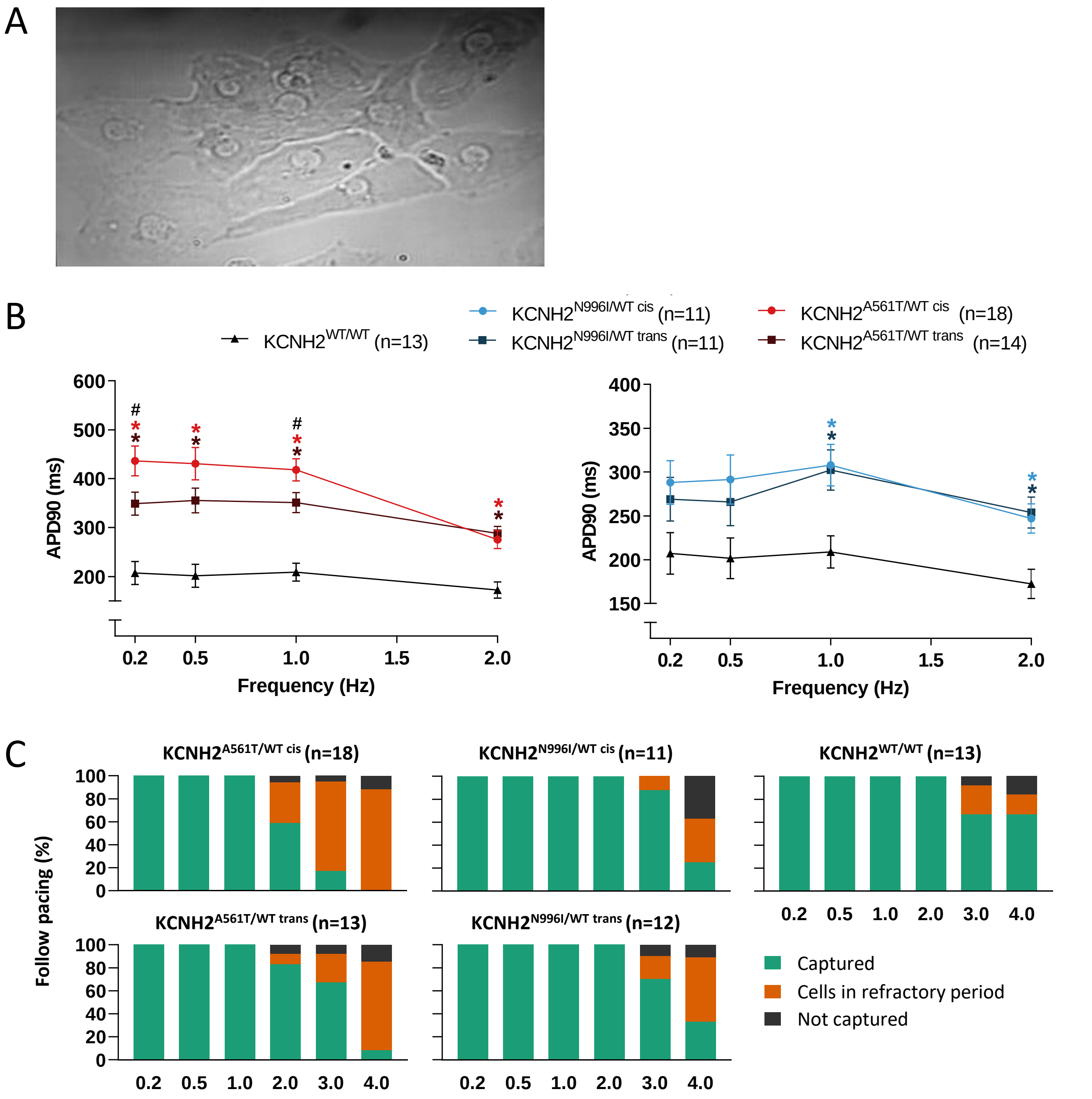

FIGURE S3 | Action potential duration (APD) and pacing responsiveness of the hiPSC-CMs from the indicated lines. (A) Example of a typical hiPSC-CM cluster measured by patch clamp. **(B)** Average APD90 for the indicated $KCNH2^{A561T/WT}$ (left panel), $KCNH2^{N996I/WT}$ (right panel), and $KCNH2^{WT/WT}$ hiPSC-CMs paced at various frequencies between 0.2-2 Hz. The data from the $KCNH2^{WT/WT}$ hiPSC-CMs are shown in both panels for comparison. * indicates statistical significance to $KCNH2^{WT/WT}$ hiPSC-CMs ($p \leq 0.01$); # indicates statistical significance between $KCNH2^{A561T/WT\ cis}$ and $KCNH2^{A561T/WT\ trans}$ hiPSC-CMs ($p \leq 0.05$); one-way ANOVA with Tukey's multiple comparisons test per indicated frequency. **(C)** Differences in the pacing responsiveness of the indicated hiPSC-CMs between 0.2-4 Hz (x-axis). hiPSC-CMs classified as 'in refractory period' could not follow the pacing frequency as they were generally still in phase 3 of repolarization when applying the stimulus. n, number of individual hiPSC-CM clusters analyzed from two or three differentiations.

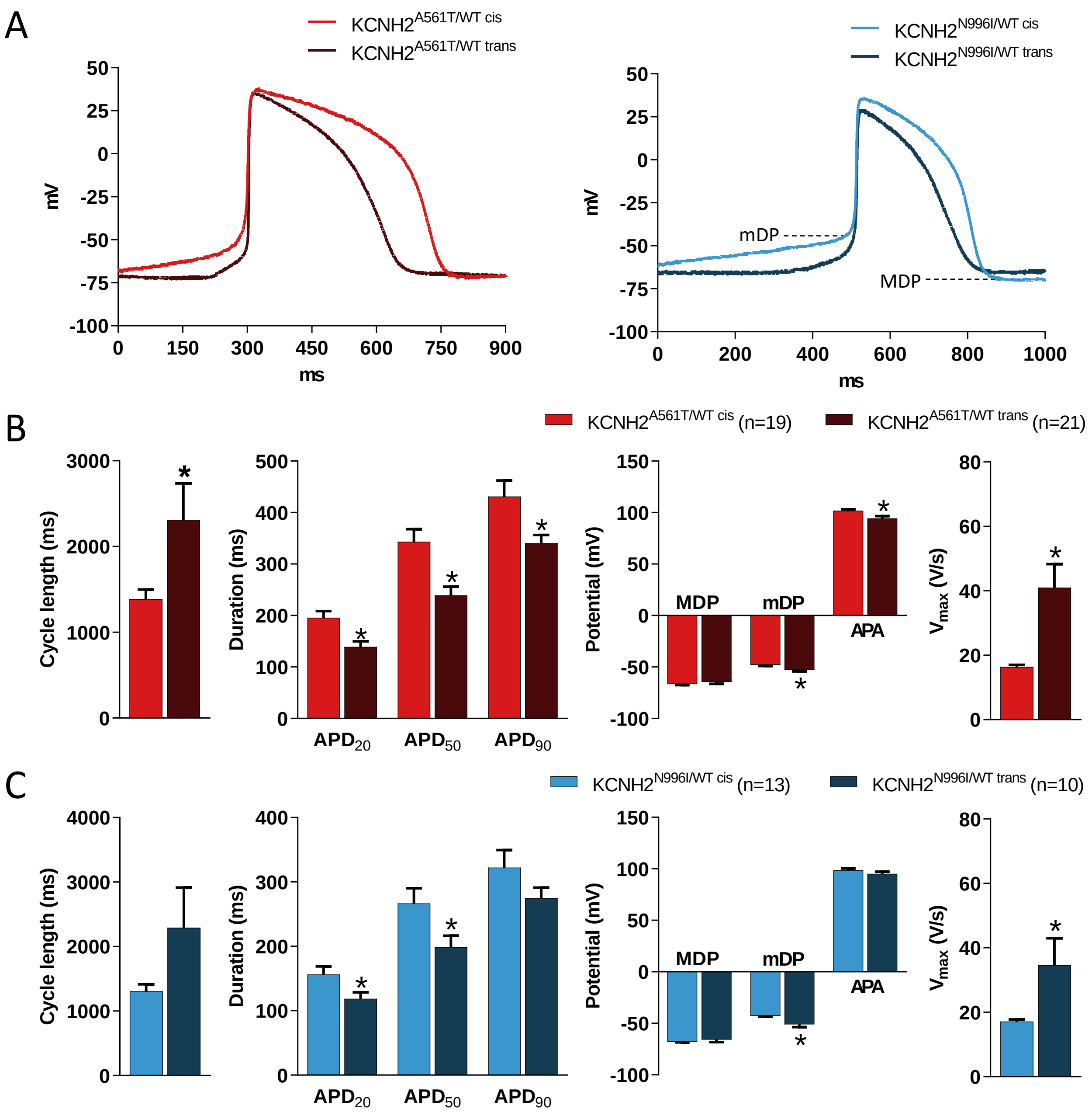

FIGURE S4 | Action potential (AP) characteristics of spontaneously beating hiPSC-CMs from the indicated lines. (A) Representative AP traces of the pairs of KCNH2$^{A561T/WT}$ (left panel) and KCNH2$^{N996I/WT}$ (right panel) hiPSC-CMs measured. Dotted lines in right panel indicate examples of MDP and mDP potentials. **(B & C)** Averaged data for the pairs of KCNH2$^{A561T/WT}$ **(B)** and KCNH2$^{N996I/WT}$ **(C)** hiPSC-CMs for cycle length, APD_{20}, APD_{50}, APD_{90}, MDP, mDP, APA, and V_{max}. n, number of individual hiPSC-CMs analyzed from two or three differentiations. * indicates statistical significance ($p \leq 0.04$; unpaired Student's t-test).

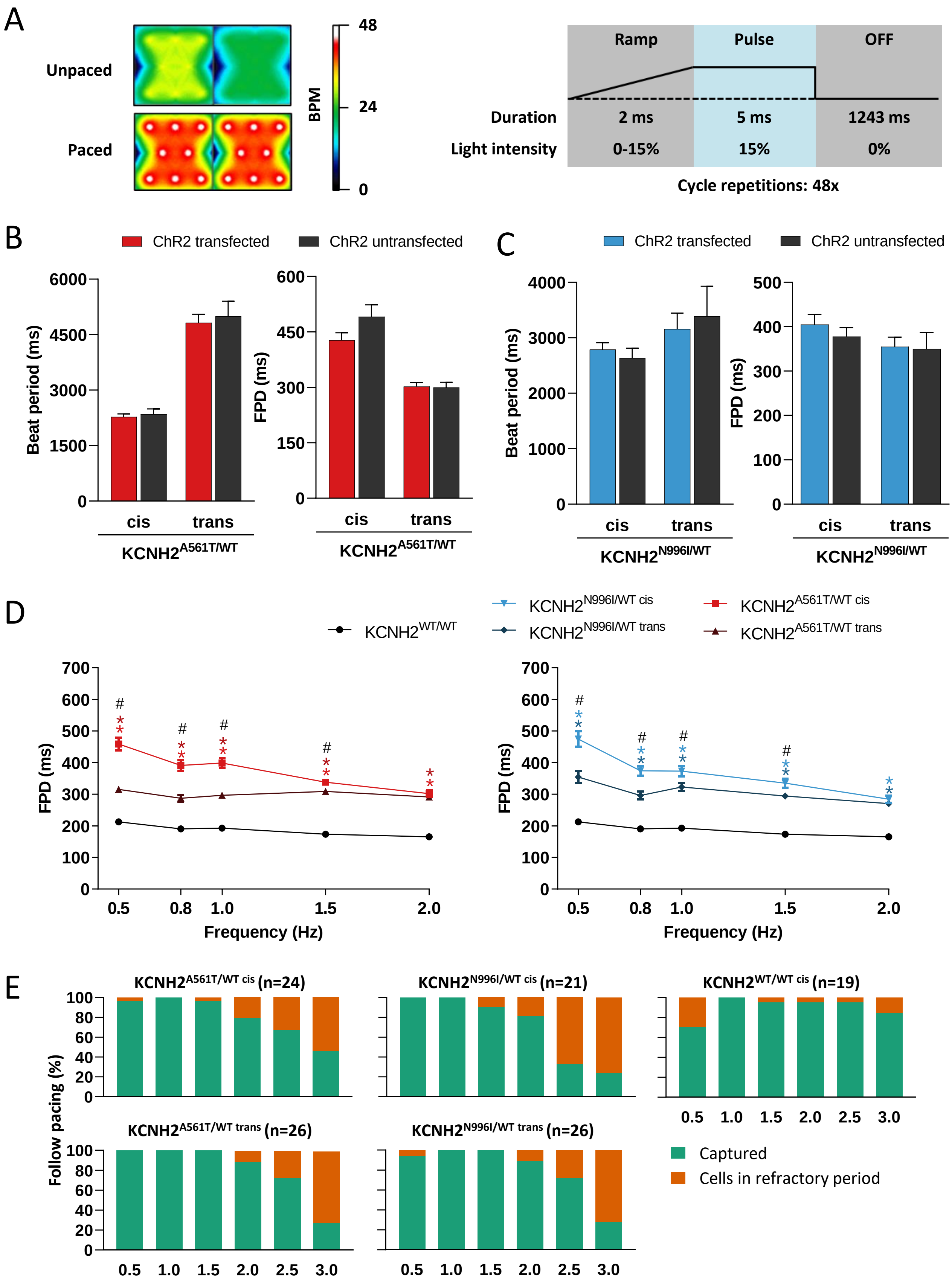
A
Unpaced
Paced
BPM
48
24
0
Ramp
Pulse
OFF
Duration
2 ms
5 ms
1243 ms
Light intensity
0-15%
15%
0%
Cycle repetitions: 48x
B
ChR2 transfected
ChR2 untransfected
Beat period (ms)
FPD (ms)
cis
trans
KCNH2A561T/WT
C
ChR2 transfected
ChR2 untransfected
Beat period (ms)
FPD (ms)
cis
trans
KCNH2N996I/WT
D
KCNH2WT/WT
KCNH2N996I/WT cis
KCNH2N996I/WT trans
KCNH2A561T/WT cis
KCNH2A561T/WT trans
FPD (ms)
Frequency (Hz)
E
KCNH2A561T/WT cis (n=24)
KCNH2N996I/WT cis (n=21)
KCNH2WT/WT cis (n=19)
KCNH2A561T/WT trans (n=26)
KCNH2N996I/WT trans (n=26)
Follow pacing (%)
Captured
Cells in refractory period

FIGURE S5 | Field potential duration (FPD) and pacing responsiveness of the hiPSC-CMs from the indicated cell lines. (A) Representative beat per minute (BPM) heatmaps for two adjacent wells of confluent KCNH2$^{N996I/WT\ trans}$ hiPSC-CMs either unpaced or paced at 0.8 Hz. The optical pacing protocol (right panel) that was applied to pace the cells at 0.8 Hz included three phases (ramp, pulse, and light off), with the cycle continuously repeated for 60 seconds. Details for all frequencies are provided in **Table S4**. **(B & C)** Average spontaneous beat period (left panel) and FPD (right panel) values for the KCNH2$^{A561T/WT}$ **(B)** and KCNH2$^{N996I/WT}$ **(C)** pairs of hiPSC-CMs untransfected or transfected with of channelrhodopsin (ChR2) [for **(B)**, transfected and untransfected respectively; KCNH2$^{A561T/WT\ cis}$ n = 31 and n = 13; KCNH2$^{A561T/WT\ trans}$ n = 31 and n = 12; for **(C)**, transfected and untransfected respectively; KCNH2$^{N996I/WT\ cis}$ n = 23 and n = 9; KCNH2$^{N996I/WT\ trans}$ n = 38 and n = 14; numbers of individual recordings from three differentiations]. **(D)** Average FPD for the indicated KCNH2$^{A561T/WT}$ (left panel) and KCNH2$^{N996I/WT}$ (right panel) pairs of hiPSC-CMs paced between 0.2-2 Hz. The data from the KCNH2$^{WT/WT}$ hiPSC-CMs are shown in both panels for comparison [KCNH2$^{WT/WT}$ n = 16-19; KCNH2$^{A561T/WT\ cis}$ n = 21-30; KCNH2$^{A561T/WT\ trans}$ n = 21-27; KCNH2$^{N996I/WT\ cis}$ n = 17-26; KCNH2$^{N996I/WT\ trans}$ n = 23-32; number of individual recordings analyzed from three differentiations]. * indicates statistical significance to KCNH2$^{WT/WT}$ ($p < 0.0001$); # indicates statistical significance between *cis* and *trans* hiPSC-CMs ($p \leq 0.03$); one-way ANOVA with Tukey's multiple comparisons test per indicated frequency. **(E)** Differences in the pacing responsiveness of the indicated hiPSC-CM monolayers to 0.5 - 3 Hz (x-axis). n, number of individual recordings analyzed from three differentiations.

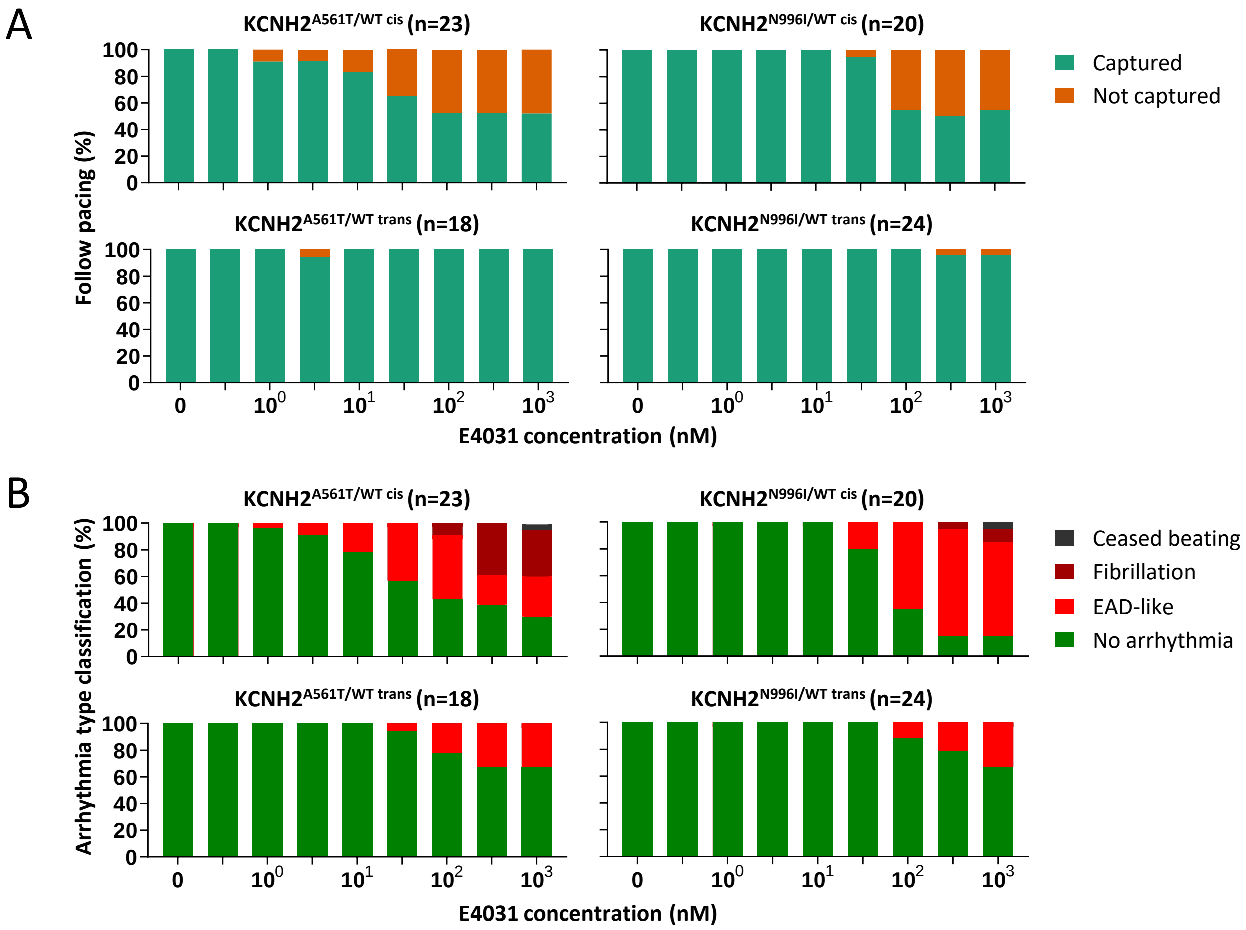

FIGURE S6 | Analysis of the effect of E4031 on KCNH2$^{A561T/WT}$ and KCNH2$^{N996I/WT}$ hiPSC-CMs with KCNH2-K897T either in *cis* or *trans* orientation. (A) Quantification of the pacing responsiveness upon increasing concentrations of E4031 (300 pM - 1 µM) for the indicated cell lines. n, number of individual recordings analyzed from three differentiations. **(B)** Assessment of the arrhythmia subtypes detected with increasing doses of E4031 (300 pM - 1 µM) for the indicated cell lines. n, number of individual recordings analyzed from three differentiations.

SUPPLEMENTARY TABLES

TABLE S1 | AP characteristics of paced (1 Hz) and spontaneous beating hiPSC-CM clusters.

Parameter	Unit	KCNH2$^{A561T/WT}$ cis	KCNH2$^{A561T/WT}$ trans	KCNH2$^{N996I/WT}$ cis	KCNH2$^{N996I/WT}$ trans
**1 Hz**					
APD_{20}	ms	181.5 ± 10.0	136.0 ± 10.3*	145.0 ± 12.8	130.0 ± 12.7
APD_{50}	ms	318.6 ± 17.3	244.0 ± 19.4*	249.3 ± 23.1	220.1 ± 21.4
APD_{90}	ms	418.2 ± 23.5	351.0 ± 21.3*	307.9 ± 24.8	302.2 ± 24.3
MDP	mV	-70.0 ± 0.9	-70.5 ± 1.7	-70.7 ± 0.9	-71.0 ± 2.3
mDP	mV	-67.3 ± 1.8	-69.5 ± 2.1	-65.9 ± 1.8	-69.4 ± 2.8
APA	mV	106.2 ± 1.4	102.0 ± 2.7	100.7 ± 1.7	100.4 ± 2.7
V_{max}	V/s	30.4 ± 5.5	44.4 ± 9.2	22.0 ± 3.4	45.7 ± 9.6*
n		18	14	11	11
**Spontaneous**					
Cycle length	ms	1383 ± 119	2311 ± 437*	1305 ± 116	2290 ± 658
APD_{20}	ms	139.1 ± 11.0	195.6 ± 13.3*	156.3 ± 13.0	118.4 ± 10.8*
APD_{50}	ms	238.9 ± 17.6	342.9 + 25.5*	266.4 + 24.9	199.0 ± 18.5
APD_{90}	ms	340.1 ± 16.7	431.0 ± 32.2	322.1 + 28.4	274.7 ± 17.6
MDP	mV	-64.6 ± 1.7	-66.5 ± 1.1	-67.7 ± 0.7	-65.8 ± 2.6
mDP	mV	-47.9 ± 1.3	-52.9 ± 1.4*	-42.5 ± 0.9	-50.9 ± 2.6*
APA	mV	94.3 ± 2.4	101.7 ± 1.5*	98.4 ± 2.0	95.2 ± 2.2
V_{max}	V/s	16.3 ± 0.7	41.0 ± 7.4*	17.0 ± 0.7	34.6 ± 8.4*
n		17	21	13	10

Values expressed as mean ± SEM. n, number of individual hiPSC-CMs analyzed from 2 or 3 independent differentiations. * indicates statistical significance between *cis-trans* hiPSC-CM pairs ($p \leq 0.04$); unpaired Student's t-test. **Abbreviations:** AP duration at 20, 50, and 90% of repolarization (APD_{20}, APD_{50}, and APD_{90}, respectively), maximum diastolic potential (MDP), minimum diastolic potential (mDP), AP amplitude (APA), maximum upstroke velocity (V_{max}).

TABLE S2 | Sequences of primers and probes used in this study.

**Purpose**	**Sequence forward primer (5' – 3')**	**Sequence reverse primer (3' – 5')**
**Primers**		
Amplification of KCNH2 exon 11-13	GTGATTGGCTAAGAGGGTGTT	GGTGGTCACAGCACTGTAGG
Sequencing of exon 11 and 13	GTGATTGGCTAAGAGGGTGTT	GGCCCTCCTTTGTTCTATGT
Amplification of KCNH2 exon 7-11	CTGATCGGGCTGCTGAAGACT	GGTGGTCACAGCACTGTAGG
Sequencing of exon 7 and 11	CTGATCGGGCTGCTGAAGACT	GGTGGTCACAGCACTGTAGG
ddPCR K897T primer	GATCACCTTCAACCTGCG	CACCTCCCCTGGCTG
ChR2 *in vitro* transcription	GAATTCTAATACGACTCACTAT AGGGAGAGGAGCCACCATGGA CTATGGC	CAGCGTATCCACATAGCGTA AAAGGAG
**Probes**		
ddPCR K897T probe (HEX conjugated)	ACG GAC +A+C+G GAC A*	
ddPCR K897K probe (FAM conjugated)	ACG GAC +A+A+G +GAC A*	

* + indicates the inclusion of locked nucleic acids to increase the specificity of the probes. **Abbreviations:** carboxyfluorescein (FAM), channelrhodopsin-2 (ChR2), droplet digital polymerase chain reaction (ddPCR), hexachlorofluorescein (HEX).

TABLE S3 | Overview of the number of replicates performed per cell line for each of the experiments.

Technique	Parameter measured	Cell format	Age* (days post thawing)	Number of replicates (# differentiations)			
				$KCNH2^{A561T/WT}$		$KCNH2^{N996I/WT}$	
				cis	*trans*	*cis*	*trans*
Flow cytometry	% (ventricular) CMs	Monolayer	7-8	4 (4)	3 (3)	5 (5)	4 (4)
ddPCR	Allelic balance	N/A	7-8	6 (2)	6 (2)	3 (1)	3 (1)
Voltage clamp	I_{Kr}	Single	7-10	19 (3)	16 (3)	18 (4)	19 (3)
Current clamp	AP	Cluster	7-12	18 (3)	14 (2)	11 (2)	11 (2)
MEA	FP, arrhythmic events	Monolayer	11-12	23 (4)	18 (3)	20 (3)	24 (4)

* All analyses were performed on hiPSC-CMs that were first cryopreserved at day 20 or 21 of differentiation. **Abbreviations:** Action potential (AP), cardiomyocytes (CMs), droplet digital polymerase chain reaction (ddPCR), field potential (FP), multielectrode array (MEA).

TABLE S4 | Settings for optical stimulation of Channelrhodopsin-2 (ChR2) messenger RNA (mRNA) transfected hiPSC-CMs using the Lumos™ system.

Frequency (Hz)	Ramp duration (ms)	Pulse duration (ms)	OFF time (ms)	Light intensity
0.5	2	5	1993	15-60%
0.8	2	5	1243	15-60%
1.0	2	5	993	15-60%
1.5	2	5	659.7	15-60%
2.0	2	5	493	15-60%
2.5	2	5	393	15-60%
3.0	2	5	326.3	15-60%
